# Mind-Body Exercises for Nurses with Chronic Low Back Pain: An Evidence-Based Review

**DOI:** 10.1155/2016/9018036

**Published:** 2016-07-03

**Authors:** Pinky Budhrani-Shani, Donna L. Berry, Patricia Arcari, Helene Langevin, Peter M. Wayne

**Affiliations:** ^1^Texas Woman's University, Nelda C. Stark College of Nursing, Houston, TX 77030, USA; ^2^Phyllis F. Cantor Center for Research in Nursing & Patient Care Services, Dana-Farber/Harvard Cancer Center, Boston, MA 02215, USA; ^3^Dana-Farber Cancer Institute, Boston, MA 02215, USA; ^4^Osher Center for Integrative Medicine, Brigham and Women's Hospital, Harvard Medical School, Boston, MA 02115, USA

## Abstract

*Background*. Chronic low back pain (CLBP) among nurses is a growing health concern. The multimodal nature of mind-body exercises has potential to impact physiological and psychological processes associated with chronic pain, affording possible advantages over conventional unimodal therapies. This paper summarizes the prevalence of and risk factors for CLBP among nurses, reviews the effectiveness in treating pain and disability of mind-body exercises (yoga and tai chi) for CLBP among the general and nursing population, and describes implications.* Methods*. Articles, published during or prior to 2015, were systematically identified through the PubMed/MEDLINE, Web of Science, and ScienceDirect databases using the following search terms:* nurses, mind-body, integrative, biopsychosocial, yoga, tai chi, back pain, *and/or* risk factors*.* Results*. Prevalence estimates of CLBP among nurses ranged from 50% to 80%. Associated risk factors for CLBP included lifestyle and physical, psychological, psychosocial, and occupational factors. No published studies were identified that evaluated yoga or tai chi for nurses with CLBP. Studies in the general population suggested that these interventions are effective in reducing pain and disability and may improve factors/processes predictive of CLBP.* Conclusion*. This review suggests that evaluating the impact of multimodal interventions such as yoga and tai chi for nurses with CLBP warrants investigation.

## 1. Introduction

Nursing is the fifth largest occupation in the United States (U.S.) and ranks as the largest of the healthcare professions [1]. Due to strenuous workloads and irregular shift schedules, musculoskeletal disorders commonly affect nurses [2–4]. The most common musculoskeletal disorder in nurses is chronic low back pain (CLBP), and this is a highly significant and growing health concern [5]. Nurses' experiences of low back pain can affect their health as well as the quality of care they provide [6]. CLBP among nurses is associated with high direct and indirect costs related to lost workdays and increased compensation claims, and CLBP often becomes a chronic health concern among nurses [7]. The nursing workforce represents an important occupational population within which to identify potential risk factors that contribute to CLBP, to identify key interventions that promote quality of life and work productivity, and to ultimately decrease the financial burden of CLBP [8].

As reviewed by Bernal and colleagues [9] using a “nonstructural” psychological and behavioral model of CLBP, even in cases where an initial injury in an anatomical structure can be identified, the pain experience of an individual will be determined by factors including previous pain experiences, beliefs and fears about low back pain, general and psychosocial health, job dissatisfaction, economic status, education, ongoing litigation, compensation claims, and social well-being. This perspective supports a shift in the focus for treating CLBP from a unimodal approach to a multimodal approach to intervention, integrating pain and disability management with physical, psychosocial, and behavioral strategies [10].

Studies of treatment options, including combinations of anti-inflammatory or other analgesic medication, muscle relaxants, directed exercise, physical therapy, education, and counseling, and treatment protocols targeting subtypes, have failed to identify an optimal treatment strategy for low back pain [11]. Not surprisingly, both patients and physicians have expressed dissatisfaction with currently available conventional treatment options and a significant number report utilizing complementary and integrative options [12]. Nonpharmacologic treatments recommended for persistent back pain include mind-body components, traditional cognitive behavioral therapy (CBT) [13], and mindfulness-based forms of CBT [14, 15]. CBT is widely recommended for patients with CLBP; however, patient access to CBT is limited [16, 17].

Additional complementary and integrative exercises such as yoga, tai chi, and qigong integrate musculoskeletal training (e.g., strength, flexibility, and coordination), breath training, and a variety of cognitive skills (e.g., body awareness, focused mental attention, and relaxation) [18]. It has been hypothesized that the multimodal nature of complementary and integrative exercises has the unique potential to target and impact multiple physiological and psychological processes associated with chronic pain conditions, thus proving to be more beneficial when compared to conventional unimodal therapies [19]. Results from the National Health Interview Survey documented that among 8196 adults who reported experiencing low back pain in the past three months, mind-body exercises were among the most commonly used complementary therapies (13%) and were rated as “very helpful” by 43% [20]. Evidence-based clinical guidelines jointly published by the American College of Physicians and the American Pain Society identified mind-body exercises as treatments for CLBP with moderate evidence of effectiveness [21].

In this review, we first summarize the evidence as it relates to the prevalence, burden, and risk factors for the development of CLBP among nurses. We then introduced the logic, rationale, and evidence supporting the use of mind-body exercises for CLBP, focusing on two popular and promising modalities, yoga and tai chi. We review the evidence for these mind-body exercises for back pain in the general population and, when available, research evidence specific to the nursing population. We conclude by discussing current gaps in knowledge and future research needs, as well as the broader implications of introducing nurses to integrative mind-body exercises.

## 2. Materials and Methods

Electronic literature searches were conducted using the PubMed/MEDLINE, Web of Science, and ScienceDirect databases from inception through January 2015. Search terms were* low back pain, nurses, risk factors, mind-body, integrative, biopsychosocial, tai chi, *and* yoga*. Searches were limited to the English language. Reference lists of retrieved articles were then examined to identify any additional references relevant for inclusion that were not captured with the online search strategy.

For studies related to prevalence, burden, and risk factors of low back pain among nurses, we included epidemiological studies, surveys, observational studies, and cross-sectional studies with the greatest emphasis placed on articles published within the last ten years. For studies evaluating mind-body exercises for low back pain among nurses, we included randomized trials, interventional studies, and observational studies. Because our systematic search resulted in no relevant studies on the impact of yoga and tai chi for nurses with CLBP, this section of our results utilizes a narrative approach for summarizing the broader literature of yoga and tai chi for low back pain.

## 3. Results and Discussion

### 3.1. Prevalence, Burden, and Risk Factors

Among healthcare personnel, nurses have the highest rate of CLBP. Among nurses worldwide, the prevalence of low back pain is 40–90% [22], and in the U.S. 52% of nurses report CLBP, with lifetime prevalence up to 80% [23, 24]. CLBP is associated with the highest rate of any occupation for workers compensation claims, and approximately 38% of nurses in the U.S. report having occupation-related back pain severe enough to require temporary work leave and 20% report changing employment due to back pain [24]. According to the 2012 U.S. Bureau of Labor Statistics, nurses with back injuries required a median of 7 days to recuperate [25]. The average compensation cost for work-related back pain was $10,698 per case in 2009 [26].

**Figure 1:**
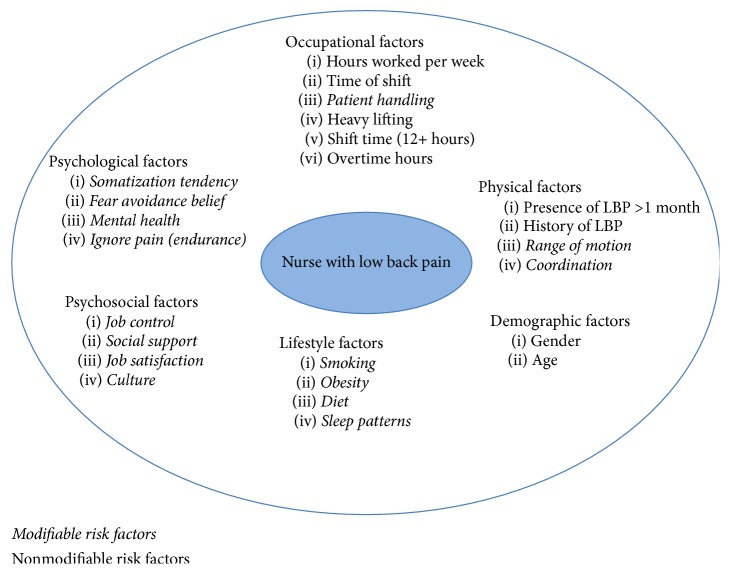
Proposed model of modifiable and nonmodifiable risk factors associated with chronic low back pain among nurses.

As mentioned, the difficulty in treating CLPB relates to the multifactorial etiology. Modifiable and nonmodifiable risk factors for CLBP among nurses (Figure 1) include demographics, lifestyle factors, occupational factors, physical factors, psychological factors, and psychosocial factors. The high physical demands associated with patient handling, longer work hours, and more demanding schedules are likely the largest contributing factors in the development of low back pain among nurses [26]. Furthermore, the aging of the nursing workforce may also contribute to this problem as the average age of an RN in the U.S. is approximately 47 years [26].

Lifestyle factors such as diet, sleep patterns, obesity, and smoking may also contribute to CLBP. Due to occupational stress, nurses frequently complained of insufficient time to eat well and exercise. Poor eating habits and exercise habits can contribute to weight gain. Results of a meta-analysis of 33 studies indicated that obesity increased the risk of low back pain and had the strongest association with seeking care for CLPB [27]. Results of the Tobacco Use Supplement-Current Population Surveys reported that the smoking rates among RNs (10.73%) and licensed practical nurses (20.55%) were higher compared to other healthcare professionals including physicians (2.31%), dentists (3.01%), and pharmacists (3.25%) [28]. Several epidemiological studies have reported an association between smoking and lumbar pain [29]. Nicotine causes vasoconstriction, which reduces the amount of oxygen and nutrients available to muscles, ligaments, and intervertebral discs, increasing chances for injuries and degenerative processes in the intervertebral discs to manifest [30]. The biological mechanisms affected by smoking that may be associated with spinal symptoms include coughing reflexes, increased fibrin deposition leading to chronic inflammation, and decreased blood flow and oxygenation of the tissues. These mechanisms affect the metabolic balance of the discs and accelerate degenerative processes leading to increased low back pain and injuries [31].

Occupational related factors for CLBP among nurses include varying shifts, shift time, hours worked per week, patient handling, and heavy lifting. Decreasing RN staff by 9% led to a 65% increase in work-related illnesses and injuries among nurses [32]. Furthermore, workday factors including working 13 or more hours per day, varying shifts, working weekends, working with less than 10 hours off between shifts, and working on previously assigned days off were significantly associated with low back pain among nurses [32]. In a systematic review of 89 studies, nursing activities were associated with an increased risk of back disorders regardless of nursing technique, personal characteristics, and non-work-related factors; patient handling conferred the highest risk of LBP among nurses [33]. Ergonomic factors including bending, carrying patients, torso twisting, and standing increased susceptibility to CLBP [34].

Psychological risk factors for LBP include fear avoidance beliefs, mental health, pain endurance, and somatization tendency [35, 36]. Results of the international Cultural and Psychosocial Influences in Disability study indicated that established psychological risk factors contribute to LBP among nurses, specifically pessimistic beliefs about pain prognosis, poor mental health, and somatization tendency [37]. In a longitudinal study of 2164 working nurses and midwives with low back pain, fear of movement, passive coping, pain severity, pain radiation, and manual handling frequency were found to significantly increase the likelihood of low back pain [8]. Pain-related fear has been hypothesized to lead to a cycle of decreased movement, connective tissue remodeling, and increased tissue stiffness. Peripheral and central nervous system sensitization may also contribute to tissue inflammation, further decreasing movement, and increasing pain-related fear and distress [8, 38].

Psychosocial risk factors for CLBP include culturally influenced health beliefs, social support, job control, and job dissatisfaction. Previous research suggests that interactions of psychosocial factors and physical exhaustion have increased the prevalence of musculoskeletal pain among nurses [39]. One prospective study found that CLBP among nurses' aides was associated with loss of support in workplace and working in night shifts [40]. A survey of 1111 nurses to examine the relationship between individual and work-related psychosocial factors and back pain indicated that low job security was associated with CLBP among nurses [12]. In addition, negative beliefs and low job satisfaction were independently associated with absence from work due to low back pain.

In summary, CLBP among nurses is a prevalent and complex problem. Multiple, interdependent risk factors contribute to the development of CLBP. In their investigative series* Injured Nurses*, the National Public Radio reported that hospitals can reduce the rate of injuries by investing in lifts, floating mattresses, and full-time coordinators to educate nurses about safe patient handling [41]. In addition, a systematic review including 83 randomized controlled trials reported that both exercise therapy and behavioral treatment were effective in reducing pain intensity at short-term follow-up for individuals with CLBP, compared to a no-treatment control group [42]. We have chosen to focus our review on mind-body exercises for low back pain.

### 3.2. Mind-Body Exercises for Low Back Pain

Increasingly, exercise is being recommended for the treatment and prevention of CLBP. A 2005 Cochrane Review identified 61 randomized controlled trials (RCTs) (43 of which were specific to CLBP) and concluded that exercise may be effective in decreasing pain and disability and improving function [43]. Interventions to date vary greatly in approach and intended effects (e.g., lumbar stabilization versus lumbar extensor strengthening exercises) [44, 45]. Reported effects of exercise often have been small, no form has been shown to be consistently better than another, and long-term adherence to exercise programs is often poor [43]. There is some evidence that interventions, which combine exercise with behavioral and/or educational components, may be more effective than individual therapies [46, 47].

Yoga and tai chi are two increasingly popular mind-body exercises that show promise for the management or treatment of low back pain [48]. Yoga originated in India over 4000 years ago and consists of physical postures (“asanas”), breathing techniques (“pranayama”), and meditation (“dhyana”) [49]. Yoga aims to improve physical and emotional balance through the use of postures and breathing techniques [50].

Tai chi (taiji, tai chi chuan, or taijiquan) is a mind-body exercise that originated in China and is growing in popularity in the U.S. [51, 52]. Tai chi, a practice based on slow intentional movements, often coordinated with breathing and imagery, aims to strengthen and relax the physical body and mind [18]. Tai chi is distinguished from yoga in its greater emphasis on dynamic postural control training and functional applications (tai chi is sometimes referred to as “yoga in motion”). Surveys have suggested that approximately 5 million Americans have practiced tai chi, and this number is increasing [51, 53–55].

### 3.3. Impact of Mind-Body Exercise on Chronic Low Back Pain

The development of CLBP from nonspecific acute pain is complex and impacts a range of physiological and psychosocial processes and symptoms. While virtually no studies are available to evaluate how mind-body exercises impact these processes in nurses with CLBP, a growing body of literature has evaluated mind-body exercises impact on relevant outcomes in other populations. Below, we first summarize the evidence on how mind-body impacts key processes known to underlie CLBP, including risk factors relevant to nurses. We then summarize the clinical outcomes of mind-body exercises for CLBP.

### 3.4. Impact of Mind-Body Exercise on Risk Factors for Chronic Low Back Pain

There are a number of modifiable risk factors particularly relevant to nurses that can be altered with mind-body exercises. The key processes listed below are particularly relevant to nurses.

#### 3.4.1. Strength and Flexibility

LBP has been associated with weakness in the lower extremities, decreased pelvic strength, and limited lower extremity and torso flexibility [56–58]. Several studies have suggested that yoga can increase muscular strength and joint flexibility among patients with low back pain [49, 59, 60]. Numerous RCTs conducted in diverse populations have demonstrated that tai chi can improve lower extremity strength and increase musculoskeletal flexibility and range of motion in the torso as well as the upper and lower extremities [61–64].

#### 3.4.2. Proprioception and Body Awareness/Sensation

Both yoga and tai chi have been hypothesized to enhance interoceptive, proprioceptive, and kinesthetic awareness [65]. Our literature search did not identify any published studies to date that have evaluated the impact of yoga on these outcomes. Recent cross-sectional and prospective studies suggest that tai chi can improve kinesthetic sense in the ankle, knee, shoulder, and hand [66–68]. Manor and colleagues [69] found that tai chi was associated with improved plantar sensation and physical function in older adults with peripheral neuropathy.

#### 3.4.3. Postural Control

Compared to healthy persons, individuals with LBP have reduced postural control [70–72]. Past studies, including RCTs and observational studies, suggested that yoga improved postural control, mobility, and gait speed among older adults [73, 74]. A systematic review of 15 studies consisting of 5 RCTs and 4 quasi-experimental, 2 cross-sectional, and 4 single-group designs found that yoga may have a beneficial effect on balance [75]. Numerous studies have also reported that tai chi reduces measures of sway (i.e., range of anteroposterior sway, range of mediolateral sway, mean radius, and swept area) as well as multiple outcomes related to balance (e.g., greater single-leg stance time and reduced fear of falling) [64, 76–78]. Other studies suggest that tai chi improves multiple aspects of gait and dynamic postural control [79–82].

#### 3.4.4. Psychological and Behavioral Factors

Individuals with low back pain often have elevated scores on questionnaires measuring pain-related fear of movement and pain-catastrophizing cognitive style [83, 84]. Other investigators have reported significant associations between low back pain and depression, anxiety, and somatization [85]. Tekur and colleagues [86] suggested that yoga can reduce pain, anxiety, and depression and improved spinal mobility in patients with low back pain more effectively than physical therapy, suggesting yoga's positive effect on psychological measures. We identified two published studies evaluating eight-week interventions of yoga exercises among nurses with stress [87–89]. Results showed that nurses had significantly lower levels of stress and significantly increased confidence in their ability to cope at treatment conclusion (8 weeks) [89] and long-term follow-up (12 months) [88]. Results of a systematic review of 37 randomized controlled studies indicated significant support for the positive effect of tai chi on a variety of psychological symptoms including anxiety, depression, stress, and fear of falling, or improving attitudes such as mood and self-esteem. In the included studies, tai chi led to significant improvements in depression compared to routine medication, education controls, waitlist controls, sham exercise controls, martial art controls, and usual daily activity controls [90]. Previous research has also suggested the positive effect of tai chi on psychosocial well-being among individuals with rheumatoid arthritis and fibromyalgia [91, 92]. Twelve weeks of tai chi reduced anxiety and depression and also improved self-efficacy, self-esteem, and motivation among individuals with rheumatoid arthritis [91]. An observational study of 28-week tai chi intervention showed improvements in anxiety, depression, self-esteem, and self-efficacy among women with fibromyalgia [92]. We are not aware of any yoga or tai chi studies to date that have specifically evaluated their impact on pain-related fear of movement or pain-catastrophizing cognitive style in patients with low back pain.

#### 3.4.5. Weight Loss

Small studies have reported yoga to be effective for weight loss among obese participants resulting in a decrease in waist circumference, hip circumference, body mass index, and total cholesterol [93, 94]. Compared to diet education or conventional exercise, limited evidence suggests tai chi may be more effective in reducing body mass index and coronary heart disease risk factors in obese individuals [95, 96]. We are not aware of any research to date that has been conducted on the impact of tai chi or yoga on weight loss among nurses or in patients with LBP.

### 3.5. Clinical Studies on Outcomes of Mind-Body Exercises in the General Population

#### 3.5.1. Yoga and Back Pain

Our literature search did not identify studies of yoga specifically targeting nurses with CLBP. However, we did identify multiple studies that have evaluated yoga for CLBP in the general population. Three systematic reviews and meta-analyses have summarized this growing body of data. Holtzman and Beggs [97] analyzed efficacy of yoga as an intervention for CLBP in 8 RCTs reporting results from 743 patients. Yoga had a significant positive effect on functional disability and pain. A second meta-analysis included ten RCTs with a total of 967 CLBP patients [98] and provided strong evidence for short-term and long-term positive effects of yoga on pain and back-specific disability. A third systematic review, including 10 clinical trials and a total of 967 participants, also concluded that there is evidence that yoga improves pain and disability in patients with CLBP [99]. While the conclusions of these three reviews generally support the potential of yoga for CLBP, it is important to note that some individual trials included in these reviews primarily reported limited or short-term benefits of yoga for CLBP [97, 98].

Two large, methodologically sound yoga trials for CLBP are briefly summarized to characterize the magnitude and scope of reported clinical effects. The first study is a three-arm parallel group stratified controlled trial of 228 adults with CLBP randomized to receive 12 weekly classes of yoga compared to conventional stretching exercises or a self-care book [100]. At the 12-week follow-up, yoga was more effective than the self-care book, but not stretching classes, in improving function and reducing symptoms due to CLBP. The second study was an RCT of 159 employees with low back pain, evaluating the cost-effectiveness of medical yoga compared with evidence-based exercise therapy and self-care advice for nonspecific low back pain. Results suggested that yoga was a cost-effective intervention as noted by differences in production loss due to fewer sickness absence days in the medical yoga group balancing the cost of implementing the medical yoga. From an employer's standpoint, medical yoga costs more than self-care advice but is beneficial due to the improved quality of life and positive incremental health benefits for employees [99]. From a societal perspective, the health gains from medical yoga are cost efficient based on a reduction in sickness absences [100, 101]. Of note, yoga appears to be safe for CLBP as reported by Sherman and colleagues [100] and further supported by conclusions in systematic reviews [97, 98].

#### 3.5.2. Tai Chi and Back Pain

As with yoga, we found no studies specifically evaluating tai chi for nurses with CLBP. Published trials of tai chi for back pain in the general population are far fewer in number. In one randomized trial [102] of 160 healthy individuals with nonspecific low back pain, tai chi was compared with a waitlisted control group. Compared to the control group, 10 weeks of tai chi reduced distress associated with back symptoms and pain intensity and improved self-report disability. Cho [103] reported that 4 weeks of tai chi was effective in reducing acute low back pain when compared to stretching among young Korean males. Two RCTs reported increased flexibility and decreased pain [104, 105]. There is compelling evidence that tai chi positively impacts multiple musculoskeletal and pain conditions other than CLBP [106–108].

We identified one published study evaluating tai chi among older female nurses with high perceived stress [109]. The design consisted of a small RCT in which 11 nurses with work-related musculoskeletal disorders were randomized to a 15-week tai chi program or a no intervention control group. The tai chi group showed significant improvements in general and mental health, greater reduction in work and general stress, and improvement in trunk flexibility compared to the control group. The tai chi group took no unscheduled time-off hours, had a 3% increase in work productivity, and had significant improvement in functional reach, whereas the control group had unscheduled absence of 49 hours during the study period. Results suggested that tai chi may be a cost effective wellness option in the workplace. With respect to safety, a systematic review of 153 randomized controlled trials of tai chi concluded that tai chi was not associated with serious adverse events but may be associated with minor musculoskeletal aches and pains [110].

### 3.6. Limitations and Future Research Directions

There are a number of limitations to this review. One limitation of this study is the broad nature of the question we ask, which intersects with many models and disciplines and thus includes terminology, which is rapidly evolving such as complementary and alternative medicine, integrative medicine, biopsychosocial, and mind-body. While our search was limited to key words such as low back pain, nurses, risk factors, integrative, and biopsychosocial, we are confident that significant trials on the key focus of our review, yoga and tai chi for CLBP in nurses, were not missed. Second, our study only included trials published in the English language. Future studies might include other languages for a better global understanding of CLBP among nurses. Finally, our review did not identify any published studies of yoga or tai chi specifically for nurses with CLBP; however, we did find compelling evidence in adult populations with CLBP that yoga and tai chi are safe and likely have positive clinical effects on disability and pain. Other studies in adults support that yoga and tai chi may positively impact multiple physical, behavioral, and psychosocial factors associated with risks of CLBP.

Future studies should be designed with sensitivity to the busy and unusual work schedules of nurses. One possibility would be worksite implementation of yoga and/or tai chi for nurses. The worksite is recognized as an appropriate setting for health promotion and disease prevention as this is where employees spend up to 60% of their day [111]. Investigators implementing a single arm study reported that a 5-minute mindfulness meditation for pediatric intensive care nurses before each work-shift was feasible and revealed significant decreases in stress from baseline to postintervention and one month following the intervention [112]. Another single arm study found that telephonic sessions of meditation led to improvement in general health, decreased stress, decreased work burnout, and improvement in several other areas among 36 nurses [113]. Results were sustained 4 months after the completion of the intervention. Furthermore, the active involvement of nurses in the planning and implementation of these programs may result in positive outcomes and sustainability of the programs. The sustainability of such programs relies on the appropriate use of data to highlight employee productivity, which may substantiate the value of the financial investment in preventive measures [114]. In a systematic review of 51 studies to determine the relationship between return on investment (ROI) and quality of study methodology in workplace health promotion programs, ROI was calculated as (benefits − costs of program)/costs of program [115]. Across all 51 studies, workplace health promotion programs indicated a 138% ROI.

Community based pragmatic trials may be a feasible design which would allow participants randomized to mind-body exercises to utilize networks of predefined schools or programs in the community which offer a variety of geographic and timing options. The convenience of pragmatic clinical trials that utilize community based mind-body training networks has been posited to enhance study participant recruitment and retention, as well as poststudy continuation of mind-body practice [116, 117].

Given the high prevalence and the complexity of the CLBP, in addition to research targeting rehabilitation and symptom management, educational initiatives that emphasize prevention should also be considered. A systematic review of work-related psychosocial risk factors and musculoskeletal disorders in nurses reported that most preventive strategies in the workplace are focused on ergonomic (proper weightlifting skills) risk factors; however, prevention should also consider reducing stress and improving the psychosocial work environment [9]. Training in nursing school might include curricula on stress management and the potential of mind-body exercises for the prevention and management of CLBP among nurses. In a survey of 342 nursing personnel, over 90% reported being interested in additional training in mind-body programs [6]. Greater than 50% of nurses were specifically interested in additional tai chi or yoga training compared to 26% and 27% for diet and exercise training, respectively.

Managing and preventing CLBP in nurses will likely enhance resilience and work engagement but may also have a positive impact on the lives that they touch through work. Advanced practice nurses are usually the first to see patients presenting with new signs of illness in the outpatient setting, and they are at the front lines and best suited to provide patients with preventive health education and recommend interventions [118]. Nurses share important relationships with patients in both inpatient and outpatient settings making them ideal candidates to make referrals for mind-body exercises and to receive training and certification themselves so they can share their knowledge with their patients.

## 4. Conclusion

CLBP among nurses is a prevalent and costly concern. To date, no studies of yoga or tai chi as an intervention for nurses with CLBP were identified. Additional research is needed to establish the efficacy of yoga and tai chi for nurses with CLBP and its potential to be implemented as part of healthcare systems. Given the safety of yoga and tai chi, the cost-effectiveness of delivering these interventions, and the outcomes of most studies suggesting their positive effects on risk factors associated with CLBP, these practices could prove to be highly beneficial for nurses. Exposing nurses to holistic mind-body exercises, both for the prevention and for rehabilitation of CLBP, has the potential to simultaneously address health concerns of both the caregivers and their patients.
